# Improving fertilization rates in IVF using rutin and quercetin in preculture medium or through oral administration

**DOI:** 10.3389/fvets.2024.1506029

**Published:** 2025-01-09

**Authors:** Hiromitsu Tanaka, Satona Ichihara

**Affiliations:** Faculty of Pharmaceutical Sciences, Nagasaki International University, Sasebo, Nagasaki, Japan

**Keywords:** flavonoid, infertility, IVF, pregnancy, quercetin, ROS, rutin

## Abstract

Fertility rates are declining in livestock such as cattle, and more than one in five Japanese couples have undergone infertility treatment or are currently infertile. Improving the fertilization rates of domesticated animals is imperative for improving their productivity and maintaining valuable lineages. In this study, the effects of rutin and quercetin on fertility and pregnancy rates were investigated by incorporating these compounds into the preculture medium for *in vitro* fertilization (IVF) or administering them orally to mice. The addition of rutin and quercetin to the preculture medium increased the IVF fertilization rate by more than twofold. Oral administration of rutin and quercetin to aged male and nulliparous female mice improved pregnancy outcomes. These findings have important implications for the non-invasive treatment of infertility.

## Introduction

Almost all dairy and beef cattle in Japan are bred through artificial insemination using frozen semen, and the fertility rates of artificially inseminated cattle have declined in recent years (1). Low fertility results in prolonged non-pregnancy periods and reduced productivity, and is therefore a critical issue that directly impacts animal husbandry profits. Infertility is defined as the inability to conceive after ≥12 months of regular sexual intercourse without the use of contraceptive methods (2), and the infertility rate of couples without children in Japan has risen to 28.2% (3). As of 2015, 18.2% of Japanese couples had reportedly undergone or were undergoing infertility testing (3). The identification of conditions that promote efficient artificial insemination or *in vitro* fertilization (IVF) would benefit human infertility treatment, animal husbandry, and the conservation of rare animal species. Research concerning infertility caused by oxidative stress has primarily been conducted using experimental animals (4). Vitamin C, vitamin E, and flavonoids are natural antioxidants. Among the flavonoids, rutin and quercetin exhibit particularly strong antioxidant activity and are now classified as vitamin-like substances, having previously been referred to as vitamin P (5). Citrus fruits, including mandarin oranges and lemons, buckwheat noodles, red wine, and green tea, are popular sources of flavonoids. Rutin is a water-insoluble polyphenol found in buckwheat, onions, and asparagus. Quercetin, the aglycone moiety of rutin, is abundant in onions, asparagus, and sunny lettuce and demonstrates antioxidant properties. Rutin and quercetin inhibit free radical-mediated oxidation through a three-stage process: superoxide anion formation, hydroxyl radical production via the Fenton reaction, and lipid peroxy radical formation (6). In previous studies, plant extracts that improve fertilization rates when included in precultures for IVF in mice were identified (7–9).

Rutin and quercetin are safe, natural compounds that have been consumed for many years and are approved for human use without restrictions on concentration (10). In this study, the effects of rutin and quercetin on sperm were evaluated using similar methods, and the impact of oral administration of EubioQuercetin (Alps Pharmaceutical Ind. Co., Ltd., Gifu, Japan) on fertility in aged mice was assessed.

## Methods

### Animals

All animal experiments were performed in accordance with the Guidelines for the Care and Use of Laboratory Animals and were approved by the Institutional Committee of Laboratory Animal Experimentation and the Research Ethics Committee of Nagasaki International University (ID no. 161). This article does not include any studies involving human subjects performed by the authors. C57BL/6 J and Institute of Cancer Research (ICR) mice were purchased from Japan SLC (Shizuoka, Japan). ICR mice were selected because they are gentle and have good breeding characteristics. The mice were provided unrestricted access to clean water and food and were housed under specific pathogen-free conditions in the animal experimentation facility of Nagasaki International University, with temperature and lighting maintained under controlled conditions throughout the experimental period.

### IVF

IVF was performed as previously described (11). Mature caudal epididymal sperm cells (~8 × 10^6^) from each mouse (10 weeks old) were incubated in 200 μL of human tubal fluid (HTF) medium (LifeGlobal, Guilford, CT, United States) without bovine serum albumin (BSA) and covered with paraffin oil. After 5 min, each sperm suspension was transferred to conditioned medium for preincubation. The control conditioned medium for sperm preincubation consisted of HTF medium supplemented with 1 mg/mL polyvinyl alcohol (PVA; Sigma, St. Louis, MO, United States) and 1.0 mM methyl-*β*-cyclodextrin (MBCD; Sigma) (12). Aliquots of 20 μL of sperm suspension in HTF medium without BSA were transferred to 20 μL of each conditioned medium containing twice the concentration of PVA, MBCD, and the test substance. The suspensions were incubated at 37°C in a humidified incubator under 5% CO_2_/95% air (motile sperm concentration: ~10,000/μL). After 50 min, 2–4 μL of sperm from each conditioned medium were used for insemination (final motile sperm concentration: 150/μL). Motile sperm swimming at the periphery of each drop were selected for insemination, as previously described (11). The test substances included rutin (Fujifilm, Tokyo, Japan), quercetin (Fujifilm), EubioQuercetin (a soluble flavonoid comprising 55% quercetin-3-*O*-rutinoside, 30% L-arginine, 3% sodium ascorbate, and 12% hydrogenated starch; Alps Pharmaceutical Ind. Co., Ltd.), sodium ascorbate (Fujifilm), and L-arginine (Fujifilm). The effect of each compound on fertility was examined during the same time period.

**Figure 1:**
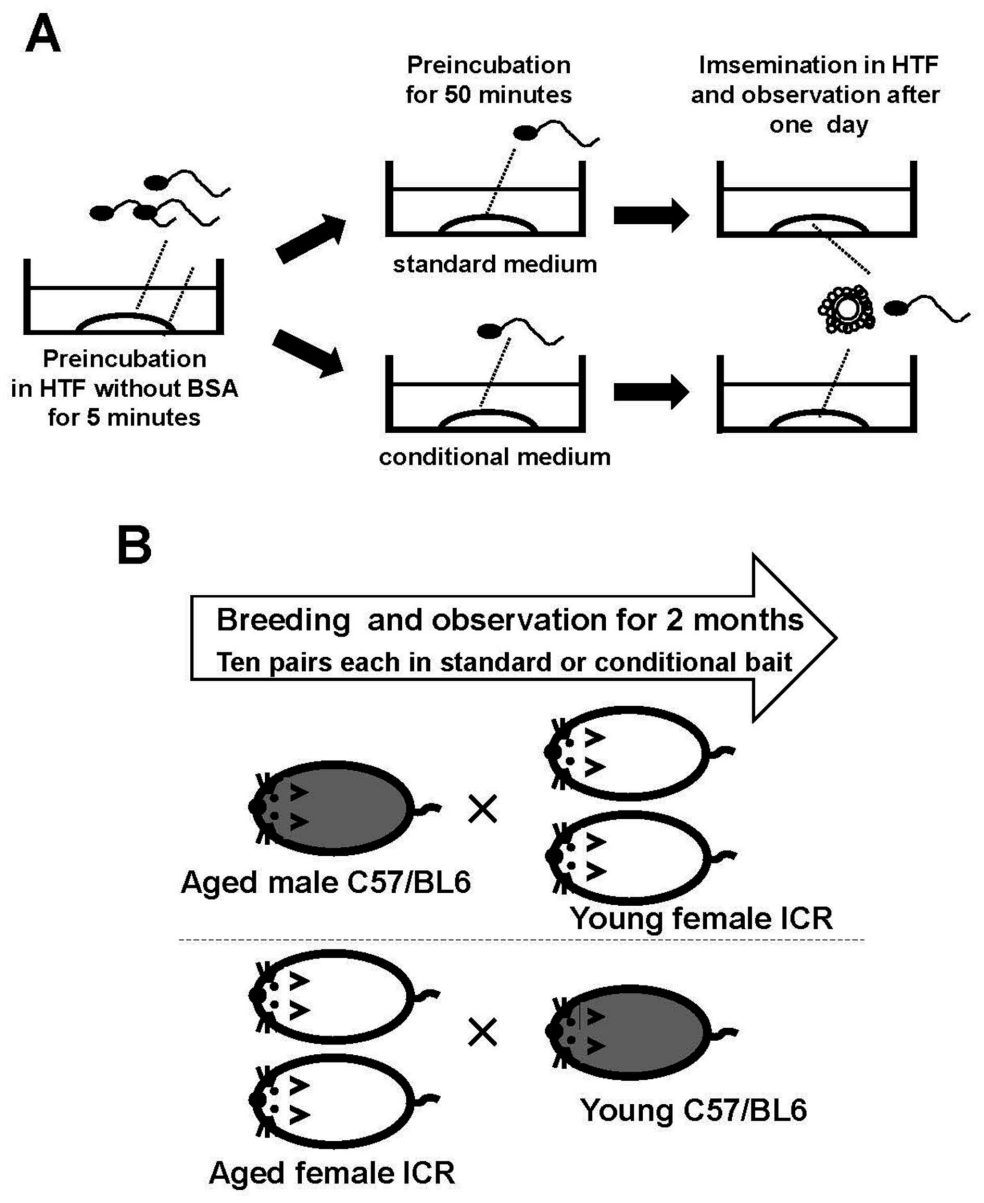
Schematic representation of the experimental procedure. Sperm from one mouse were incubated in HTF medium without BSA for 5 min to allow the sperm clumps to disperse. The sperm were then transferred to modified HTF under specific conditions to activate them before use in insemination **(A)**. Aged mice underwent multiple pregnancies. One aged male mouse was mated with two young females, and two aged female mice were mated with one young male. The male mice belonged to the C57/BL6 strain, and the female mice belonged to the ICR strain. Pregnancies of 10 pairs of mice were monitored **(B)**.

Female mice (10 weeks old) were superovulated by intraperitoneal injection of 5 IU pregnant mare serum gonadotropin (Asuka Inc., Tokyo, Japan) at 18:00, followed 48 h later by 5 IU of human chorionic gonadotropin (Asuka Inc.). Mice were euthanized 14 h after the second injection. Cervical dislocation was performed immediately before the experiment. Ovaries with oviducts were transferred to 30 mm dishes containing 6 mL of paraffin oil (Nacalai Tesque, Kyoto, Japan) without medium. Cumulus–oocyte complexes were collected from the ampullae of the uterine tubes (not the uterus) and transferred under a stereomicroscope to 200 μL drops of HTF medium covered with paraffin oil. Four to six cumulus–oocyte complexes were placed in each 200 μL drop of HTF medium for insemination. A sperm suspension cultured in conditioned medium was added to the insemination drops (Figure 1A). At 24 h after insemination, the fertilization rate was determined as the proportion of two-cell-stage embryos among all oocytes.

### Oral administration

The mice were provided food and water *ad libitum*. The diet consisted of 500 N Hi-Durability IRRD M/R (Japan SLC, Shizuoka, Japan). Bait containing the test substance was prepared by mixing the test substance with Hi-Durability IRRD M/R, as previously described (13). Based on the average daily consumption of 5 g of bait by the mice and referring to a previous mouse experiment (13), 6 mg of rutin or quercetin were added to 5 g of Hi-Durability IRRD M/R. The 500 N Hi-Durability IRRD M/R was moistened with hot water at 50°C and combined with the test substance. The mixture was shaped into clumps with a diameter of 3 cm, which were dried overnight at 80°C. To prepare bait containing EubioQuercetin, 6 mg of EubioQuercetin were sprinkled onto lightly moistened Hi-Durability IRRD M/R, which was then dried at room temperature before being fed to the mice. Each mouse ingested about 6 mg (200 mg/kg body weight) of rutin, quercetin, or EubioQuercetin per day.

Male C57BL/6 mice aged ≥1 year were paired with two 8-week-old female ICR mice, forming 20 mating pairs. The gestation period was 19 days. Ten pairs were fed bait containing the test substance, and the other ten pairs were fed the standard diet. Female mice were observed for pregnancy over a 2-month period. Additionally, two female ICR mice aged ≥8 months were paired with 8-week-old male C57BL/6 mice to form 20 pairs. These pairs were divided into two groups: 10 pairs were fed bait containing the test substance, whereas the remaining 10 pairs received the standard diet for 2 months (Figure 1B).

### Statistical analysis

Data are expressed as the mean ± standard deviation. Statistical analysis was performed using Student’s *t*-test and one-way analysis of variance with Dunnett’s or Tukey’s *post hoc* tests. In all analyses, *p* < 0.05 was considered statistically significant.

## Results

**Figure 2:**
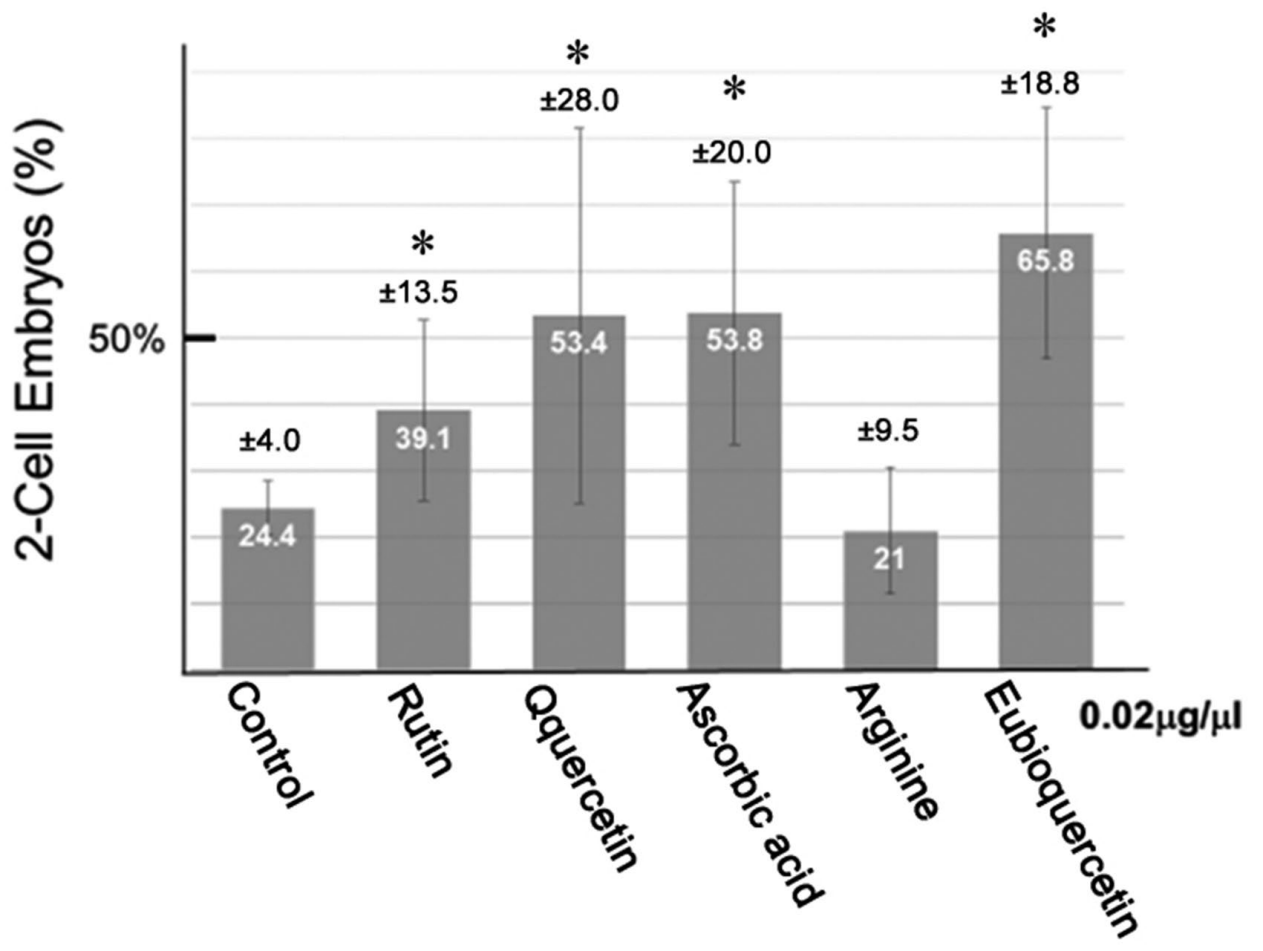
Effects of flavonoids on the *in vitro* fertilization (IVF) rate. Sperm from aged BALB/cByJJcl mice were preincubated in conditioned medium containing various compounds (0.02 mg/mL). Fertilization rates varied among aged BALB/cByJJcl mice. Asterisks indicate significant differences in fertilization rates (*n* = 5, *p < 0.05).

**Figure 3:**
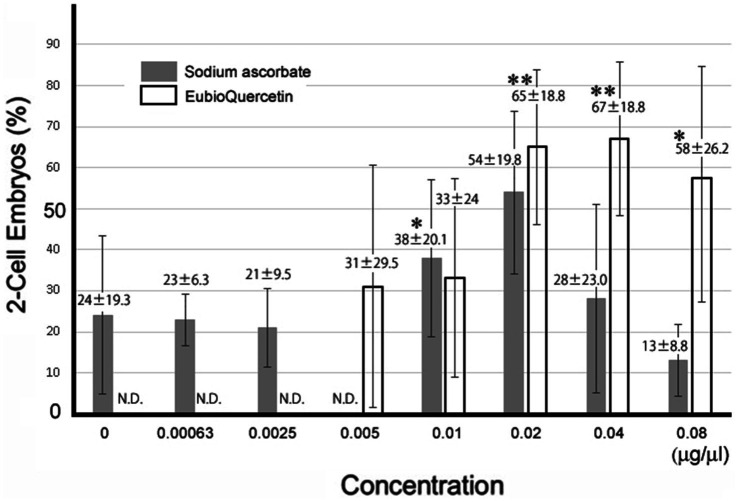
Optimal concentrations of sodium ascorbate and EubioQuercetin for IVF. Sperm from aged BALB/cByJJcl mice were preincubated in conditioned medium containing various compounds at different concentrations. Asterisks indicate significant differences in fertilization rates (*n* = 5, * *p* < 0.05, ** *p* < 0.01).

**Figure 4:**
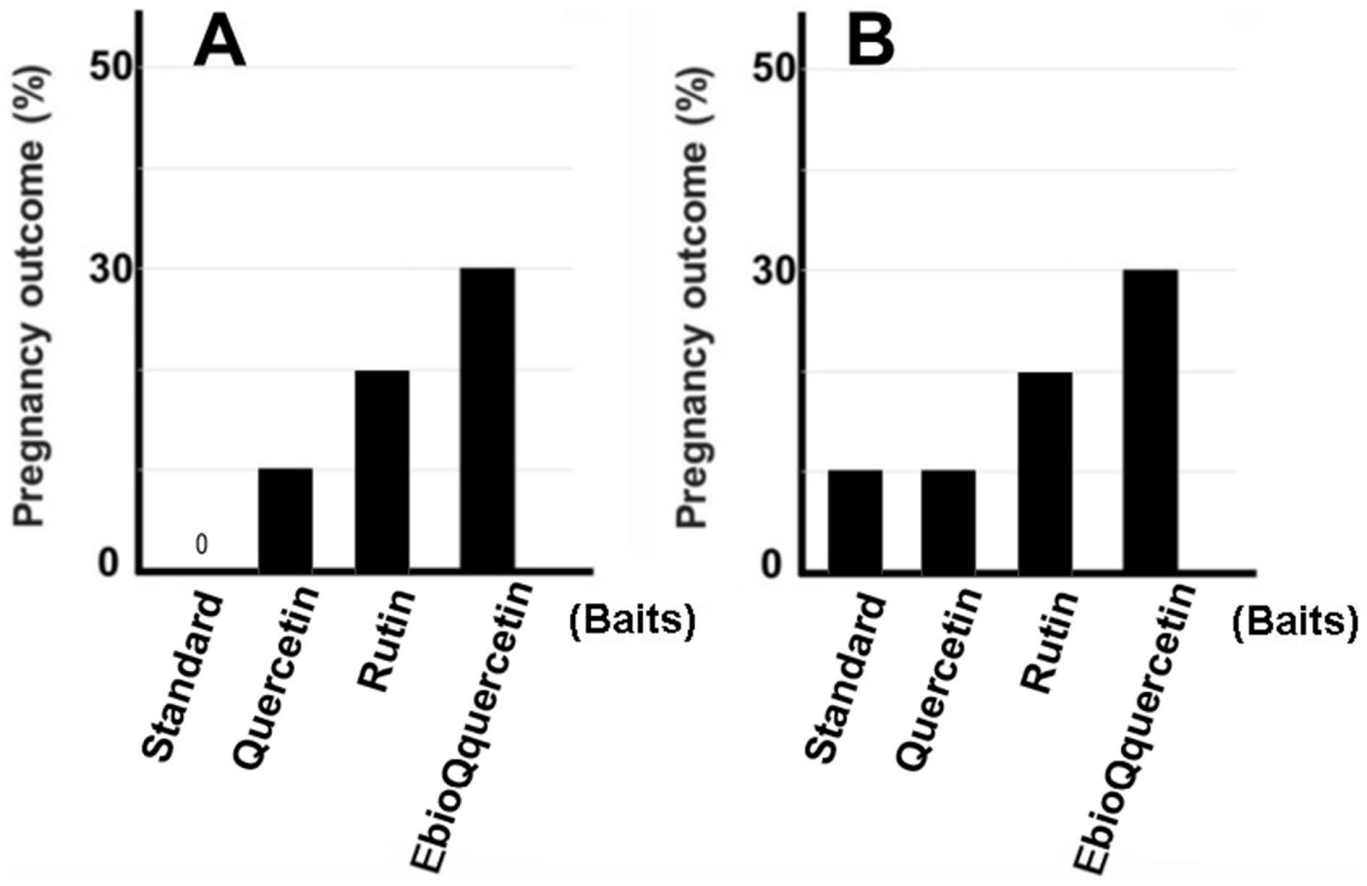
Pregnancy rate in aged mice fed EubioQuercetin. **(A)** Male mice aged ≥1 year, fed EubioQuercetin for 2 months, were mated with two 2-month-old female mice. **(B)** Two 8-month-old female mice were mated with one 3-month-old male mouse. Ten of the 20 pairs of mice that produced no offspring within 2 months were subsequently fed EubioQuercetin for 2 months.

In previous studies, we identified plant extracts that improved IVF fertilization rates in mice when added to precultures (7–9). In this study, the effects of rutin and quercetin on fertility were evaluated using similar methods. The effect of each compound on fertility was examined during the same time period. Rutin, quercetin, and EubioQuercetin significantly improved fertilization rates (Figure 2). Sodium ascorbate, a component of EubioQuercetin, also significantly enhanced fertility, whereas L-arginine, another component, did not affect fertilization rates. Next, we explored the optimal effective concentrations of EubioQuercetin and sodium ascorbate for enhancing fertility. The optimal concentration of EubioQuercetin was ≥0.04 mg/mL (Figure 2), whereas the optimal concentration of sodium ascorbate was 0.02 mg/mL; higher concentrations reduced fertility. High concentrations of ascorbic acid may have unfavorable localized effects on sperm function. The sodium ascorbate concentration in the medium was 0.00063 mg/mL when EubioQuercetin was added at 0.02 mg/mL. No improvement in fertility was observed with sodium ascorbate at this low concentration. The highest fertilization rate was observed with EubioQuercetin, likely due to the synergistic effects of rutin and ascorbic acid (Figure 3). Subsequently, we evaluated the effects of orally administered rutin, quercetin, and EubioQuercetin on fertility in aged mice. Pregnancy was confirmed in 10, 20, and 30% of females paired with older males consuming a diet supplemented with quercetin, rutin, and EubioQuercetin, respectively (Figure 4A). Additionally, two female ICR mice aged ≥8 months were crossed with 8-week-old male C57BL/6 mice. Pregnancy was confirmed in 10, 20, and 30% of older females consuming a diet supplemented with quercetin, rutin, and EubioQuercetin, respectively (Figure 4B). The pregnancy rate of older female mice fed the standard diet was 10% (Figure 4B). Precise differences among the effects of the test substances on fertility improvement could not be determined, but oral administration of the vitamin-like substance quercetin and its glycosides clearly influenced pregnancy rates in older female mice.

## Discussion

Rutin and quercetin have been shown to exert a diverse range of effects in animal experiments (5). Quercetin has been reported to enhance antioxidant activity, reduce lipid peroxidation and oxidative stress, prevent proapoptotic gene expression, increase testosterone levels, and facilitate spermatogenesis (14). Spermatogenesis declines with age in humans and domestic animals, leading to infertility (15, 16). Reactive oxygen species (ROS) have been reported to negatively affect ejaculated sperm and fertilization (17), and it is plausible that mature sperm are more vulnerable to ROS in aging males. Oocyte and ovum maturity also decline with age, contributing to infertility (5). These results suggest that the compound directly affects sperm during *in vitro* fertilization. Because sperm exist in the extracellular environment, they are particularly sensitive to ROS in the surrounding medium. Rutin and quercetin have been shown to reduce sperm ROS levels (18). Although we did not observe a decrease in ROS levels, ROS in the medium may have contributed to the improved fertilization rate.

Quercetin, a lipid-soluble small molecule, is passively taken up into cells, which may enhance its ability to scavenge metabolic ROS generated during sperm activity (5). The increase in fertility observed in this study was highest with EubioQuercetin and lowest with rutin. These results suggest differences in molecular stability and cellular uptake efficiency. EubioQuercetin is a formulation designed to stabilize quercetin and increase its water solubility, thus improving its cellular uptake. Although quercetin is unstable in culture medium, it may be taken up into cells in a concentration-dependent manner. The aglycone of rutin may also be taken up into cells during its decomposition process.

Quercetin is reportedly incorporated into extracellular vesicles (EVs) and efficiently taken up by cells via EVs (19–21). Active material exchange through EVs has been observed in sperm (22, 23). During sperm preincubation, quercetin may be taken up by sperm via EVs. Although quercetin is readily chemically decomposed in aqueous solution, encapsulation in EVs may enhance its stability and cellular uptake.

Both of these natural, low-molecular-weight compounds are easy to administer orally and were shown to improve fertility in aged mice in this study. EubioQuercetin, an easily absorbed preparation, was particularly effective at improving fertility. Oral intake of quercetin may also improve libido in both males and females by increasing vitality (5). It is unclear whether the oral administration of rutin and quercetin directly or indirectly influenced germ cell differentiation. Although pregnancy was occasionally observed in mice fed the standard diet, the total number of offspring per litter tended to be low (data not shown). Quercetin is known to pass through the placenta (24). Although it remains unknown whether the amount of food taken orally in this study had a beneficial effect on embryo development, our findings demonstrate that it did not have a detrimental effect. Additionally, it may have directly or indirectly protected aged oocytes (25). Quercetin is known to act on the placenta. In this experiment, oral intake continued during pregnancy, suggesting that quercetin exerted physical effects or acted on the placenta to support pregnancy continuation, which might otherwise have been compromised due to advanced maternal age (25, 26).

In humans, lifestyle factors such as diet, smoking, and environmental stress, which were not included in our experimental design, are cited as factors contributing to infertility. However, studies on human quercetin intake have identified a number of therapeutic functions (5, 14, 27). As the present study showed efficacy in humans at safe intake levels, we anticipate that this finding may be applied to other animals. Because the results of our oral intake experiment on mice are difficult to apply directly to human food intake, which occurs via different methods, future studies should consider the dosage and administration methods for other animals, including humans. Further evaluation of the kinetics and molecular mechanisms of rutin and quercetin would be valuable for understanding and improving animal infertility.

## Conclusion

Rutin and quercetin increased the fertility rate when added to IVF medium, and their oral administration improved fertility in aged mice.

## Data Availability

The raw data supporting the conclusions of this article will be made available by the authors, without undue reservation.

## References

[1] Endo N. Possible causes and treatment strategies for the estrus and ovulation disorders in dairy cows. J Reprod Dev. (2022) 68:85–9. doi: 10.1262/jrd.2021-125, PMID: 35153250 PMC8979803

[2] World Health Organization (WHO). Infertility prevalence estimates, 1990–2021. Geneva (2023). Available at: https://www.who.int/publications/i/item/978920068315 (accessed December 19, 2024).

[3] 15th Basic Survey on Birth Trends. National Institute of population and social security research: IPSS. (2017). Available at: https://www.ipss.go.jp/ps-doukou/e/doukou15/Nfs15R_points_eng.pdf (accessed September 1, 2024).

[4] Zargari F Rahaman MS KazemPour R Hajirostamlou M. Arsenic, oxidative stress and reproductive system. J Xenobiot. (2022) 12:214–22. doi: 10.3390/jox12030016, PMID: 35893266 PMC9326564

[5] Chen S Tang Y Gao Y Nie K Wang H Su H. Antidepressant potential of quercetin and its aglycon derivatives: a comprehensive review and update. Front Pharmacol. (2022) 13:865376. doi: 10.3389/fphar.2022.865376, PMID: 35462940 PMC9024056

[6] Afanas'ev IB Dorozhko AI Brodskii AV Kostyuk VA Potapovitch AI. Chelating and free radical scavenging mechanisms of inhibitory action of rutin and quercetin in lipid peroxidation. Biochem Pharmacol. (1989) 38:1763–9. doi: 10.1016/0006-2952(89)90410-3, PMID: 2735934

[7] Tung NH Shoyama Y Wada M Tanaka H. Two activators of in vitro fertilization in mice from licorice. Biochem Biophys Res Commun. (2015) 467:447–50. doi: 10.1016/j.bbrc.2015.09.088, PMID: 26392313

[8] Ohta T Uto T Shoyama Y Sakyiamah MM Appiah AA Tanaka H. *In vitro* fertilization using sperm activated by ML-2-3 isolated from Morinda lucida Bentham leaves. Reprod Med Biol. (2022) 21:e12455. doi: 10.1002/rmb2.12455, PMID: 35414765 PMC8986972

[9] Aoki Y Tsujimura A Nagashima Y Hiramatsu I Uesaka Y Nozaki T. Effect of *Lepidium meyenii* on in vitro fertilization via improvement in acrosome reaction and motility of mouse and human sperm. Reprod Med Biol. (2018) 18:57–64. doi: 10.1002/rmb2.12251, PMID: 30655722 PMC6332831

[10] Ishikura Y Fuji W Sakakibara Y Sakano K Hayashi M Ebihara S. Safety evaluation of excessive intake of the green tea beverage containing quercetin glycoside (enzymatically modified isoquercitrin)in healthy adults include obesity persons. Jpn Pharmacol Ther. (2012) 40:505–12.

[11] Tung NH Shoyama Y Wada M Tanaka H. Improved in vitro fertilization ability of mouse sperm caused by the addition of licorice extract to the Preincubation medium. Open Reprod Sci J. (2014) 6:1–7. doi: 10.2174/1874255601406010001

[12] Takeo T Hoshii T Kondo Y Toyodome H Arima H Yamamura KI. Methyl-Beta-Cyclodextrin improves fertilizing ability of C57BL/6 mouse sperm after freezing and thawing by facilitating cholesterol efflux from the cells. Biol Reprod. (2008) 78:546–51. doi: 10.1095/biolreprod.107.065359, PMID: 18046011

[13] Tanaka H Matsushita H Tokuhiro K Fukunari A Ando Y. Ingestion of soybean sprouts containing a HASPIN inhibitor improves condition in a mouse model of Alzheimer's disease. Biology (Basel). (2023) 12:320. doi: 10.3390/biology12020320, PMID: 36829593 PMC9953708

[14] Hosseinabadi F Faraji T Malmir M. Impact of quercetin on sperm parameters, testicular tissue, and sex hormone a systematic review. Jorjani Biomed J. (2021) 9:33–54. doi: 10.52547/jorjanibiomedj.9.4.33, PMID: 38904341

[15] Yen CA Curran SP. Incomplete proline catabolism drives premature sperm aging. Aging Cell. (2021) 20:e13308. doi: 10.1111/acel.13308, PMID: 33480139 PMC7884046

[16] Virant-Klun I Imamovic-Kumalic S Pinter B. From oxidative stress to male infertility: review of the associations of endocrine-disrupting chemicals (bisphenols, phthalates, and parabens) with human semen quality. Antioxidants (Basel). (2022) 11:1617. doi: 10.3390/antiox11081617, PMID: 36009337 PMC9405245

[17] Rodríguez-González GL Reyes-Castro LA Vega CC Boeck L Ibáñez C Nathanielsz PW. Accelerated aging of reproductive capacity in male rat offspring of protein-restricted mothers is associated with increased testicular and sperm oxidative stress. Age (Dordr). (2014) 36:9721. doi: 10.1007/s11357-014-9721-5, PMID: 25354645 PMC4213342

[18] Salehi E Shadboorestan A Mohammadi-Bardbori A Mousavi A Kargar-Abargouei E Sarkoohi P. Effect of crocin and quercetin supplementation in cryopreservation medium on post-thaw human sperm quality. Cell Tissue Bank. (2024) 25:531–40. doi: 10.1007/s10561-023-10110-3, PMID: 37776436

[19] Cui Z Zhao X Amevor FK du X Wang Y Li D. Therapeutic application of quercetin in aging-related diseases: SIRT1 as a potential mechanism. Front Immunol. (2022) 13:943321. doi: 10.3389/fimmu.2022.943321, PMID: 35935939 PMC9355713

[20] Yin D Cao JY Yang Y Li ZT Liu H Tang TT. Quercetin alleviates tubulointerstitial inflammation by inhibiting exosomes-mediated crosstalk between tubular epithelial cells and macrophages. Inflamm Res. (2023) 72:1051–67. doi: 10.1007/s00011-023-01730-2, PMID: 37039838

[21] Sergazy S Zhetkenev S Shulgau Z Chulenbayeva L Kamyshanskiy Y Nurgaziyev M. Investigating the suitability of Mare's Milk-derived exosomes as potential drug carriers. Biomol Ther. (2024) 14:1247. doi: 10.3390/biom14101247, PMID: 39456180 PMC11506534

[22] Martínez-Díaz P Parra A Montesdeoca M Barranco I Roca J. Updating research on extracellular vesicles of the male reproductive tract in farm animals: a systematic review. Animals (Basel). (2024) 14:3135. doi: 10.3390/ani14213135, PMID: 39518859 PMC11545059

[23] Panner Selvam MK Chandra PK Bakhtiary Z Busija DW Sikka SC. Untargeted Metabolomic profiling of extracellular vesicles isolated from human seminal plasma. Biomol Ther. (2024) 14:1211. doi: 10.3390/biom14101211, PMID: 39456146 PMC11506783

[24] Biechonski S Gourevich D Rall M Aqaqe N Yassin M Zipin-Roitman A. Quercetin alters the DNA damage response in human hematopoietic stem and progenitor cells via topo II- and PI3K-dependent mechanisms synergizing in leukemogenic rearrangements. Int J Cancer. (2017) 140:864–76. doi: 10.1002/ijc.30497, PMID: 27813122

[25] Jahan S Abid A Khalid S Afsar T Qurat-Ul-Ain Shaheen G. Therapeutic potentials of quercetin in management of polycystic ovarian syndrome using Letrozole induced rat model: a histological and a biochemical study. J Ovarian Res. (2018) 11:26. doi: 10.1186/s13048-018-0400-5, PMID: 29615083 PMC5883607

[26] Yoshida K Kusama K Shinohara G Sato S Yoshie M Tamura K. Quercetin stimulates trophoblast fusion via the mitochondrial function. Sci Rep. (2024) 14:287. doi: 10.1038/s41598-023-50712-1, PMID: 38168580 PMC10762005

[27] Baqer SH Al-Shawi SG Al-Younis ZK. Quercetin, the potential powerful flavonoid for human and food: a review. Front Biosci (Elite Ed). (2024) 16:30. doi: 10.31083/j.fbe1603030, PMID: 39344383

